# Current practice and the role of the CT in the management of penetrating liver injuries at a Level I trauma center

**DOI:** 10.4103/0974-2700.76838

**Published:** 2011

**Authors:** Beat Schnüriger, Peep Talving, Raffaella Barbarino, Galinos Barmparas, Kenji Inaba, Demetrios Demetriades

**Affiliations:** Division of Acute Care Surgery and Surgical Critical Care, Los Angeles County Medical Center, University of Southern California, Los Angeles, CA, USA

**Keywords:** Abdomen, computed tomography, gunshot wounds, nonoperative management, penetrating liver injury, stab wounds, trauma

## Abstract

**Background:**

The liberal utilization of computed tomography (CT) has significantly contributed to overall improvements in trauma care. However, the role and the current practice of the CT examinations in the management of patients with penetrating liver injuries are scantily documented.

**Aims:**

This study was aimed to assess the current practice and the role of the admission and follow-up CT in patients with penetrating liver injuries.

**Setting and Design:**

This is a retrospective study at a Level I trauma center. Study period is from 01/2005 to 12/2007.

**Methods:**

All patients with penetrating liver injuries were analyzed.

**Results:**

Overall, 178 patients with penetrating liver injuries were assessed. A total of 123 (69.1%) patients underwent emergent laparotomy without preoperative CT due to signs of peritonitis (47.8%), hypotension (16.3%), or a GCS of ≤8 (5.1%). In this group of patients, no nontherapeutic laparotomy occurred. The remaining 55 (30.9%) patients underwent CT scan evaluation on admission. Of these, 54.5% were selected for nonoperative management (NOM). Sensitivity and specificity of the admission CT to predict a positive laparotomy was 95.7% and 90.6%, respectively. Overall, 80.6% of isolated liver injuries were successfully managed nonoperatively. Thirty-three (18.5%) patients died within 72 h. In the remaining 145 patients, 33 liver-related complications occurred in 17.2% (25 of 145) of patients.

**Conclusion:**

Two-thirds of patients with penetrating liver injuries require emergent laparotomy, mainly due to associated injuries. The remaining one-third of patients, however, is amenable for an admission CT, which reliably predicts successful NOM. Moderate or severe injuries require follow-up CT because of the high incidence of asymptomatic liver-related complications.

## INTRODUCTION

In hemodynamically stable, evaluable patients without signs of peritonitis, selective nonoperative management (NOM) of penetrating liver injury (LI) has been shown to be feasible and safe.[1–8] Even high-grade injuries can be safely selected for NOM at centers with extensive experience in penetrating injuries.[2–4,8]

The liberal utilization of intravenous contrast-enhanced computed tomography (CT) has significantly contributed to overall improvements in trauma care, and has been associated with an increased use of NOM in penetrating liver injuries.[2,8,9] However, the experiences with the admission CT in patients sustaining a penetrating LI are limited, because the majority of these patients are not amenable for a preoperative advanced imaging. While it is important to assess the value of the initial CT scan to predict a successful NOM, the role and current practice of the follow-up CT among these patients also requires further investigation. There is evidence suggesting that in moderate-to-high-grade injuries, a follow-up CT should be performed and in low-grade blunt liver injuries follow-up CT scan is not mandatory.[10,11]

The purpose of this study was to delineate the current practice of CT utilization at a large Level 1 trauma center in victims of penetrating liver injuries and to examine the role of the admission CT and follow-up CT in altering further management.

## METHODS

After IRB approval, the Los Angeles County + University of Southern California Medical Center (LAC + USC) trauma registry was queried and all admitted patients sustaining a LI due to a penetrating mechanism [gunshot wound (GSW) or stab wound (SW)] during the years 2005–2007 were identified using the International Classification of Diseases–9^th^ Edition (ICD-9) codes 864.0-9. At LAC + USC Medical Center, all trauma patients are captured and entered in real time into the institutional trauma registry. Patient data were collected using a computerized spreadsheet (Microsoft Excel 2003, Microsoft Corporation, Redmond, WA) and included demographics and injury characteristics on admission including systolic blood pressure (SBP), Glasgow Coma Scale (GCS) score, and Injury Severity Score (ISS). All operative reports were reviewed and intraoperative findings were documented. In addition, the final attending radiologist’s CT-reports and dates of the initial and follow-up CT-investigations were reviewed.

Liver injuries were graded using the American Association for the Surgery of Trauma-Organ Injury Scale I (AAST-OIS 1).[12] The grade of each injury was determined either from the initial CT (PQ 5000 or 6000; Picker International, Cleveland, OH) or from the operative report of patients who were taken emergently to the operating room (OR) prior to obtaining a CT. Grades I and II were grouped as low-grade injuries, grade III injuries were classified as moderate, and grades IV and V as high-grade injuries.

Values are reported as means ± standard deviation (SD) or percentages. *P* values were obtained from χ^2^ - or Fischer’s exact test for proportions and from *t*-test or Mann–Whitney test for means. All statistical analysis was performed using the Statistical Package for Social Sciences (SPSS Windows^©^), version 12.0 (SPSS Inc., Chicago, IL).

As per protocol, all clinically evaluable patients with penetrating injuries to the abdomen, who are hemodynamically stable and have no signs of peritonitis, undergo a contrast-enhanced CT scan evaluation of the abdomen under close hemodynamic monitoring in the radiology suite. Patients with radiological signs of hollow viscus perforation or those who develop subsequent hemodynamic instability or peritonitis undergo exploratory laparotomy. The severity of the LI is not considered a contraindication for NOM.

All patients with grades III and IV penetrating liver injuries undergo a follow-up CT scan evaluation a few days after admission.

## RESULTS

**Figure 1:**
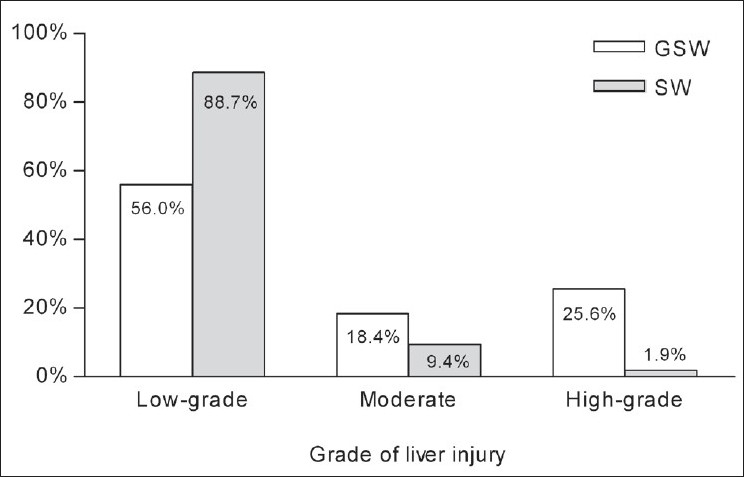
Distribution of the grades of liver injury according to mechanism. GSW: Gunshot wound; SW, stab wound

During the 3-year study period, 178 patients with penetrating liver injuries were admitted, 125 (70.2%) due to GSW and 53 (29.8%) due to SW. The epidemiology and clinical findings on admission are shown in Table 1. GSW were significantly more likely to cause moderate-to-severe liver injuries than SW (44.0% vs. 11.3%, *P* < 0.001) [Figure 1].

**Table 1:** Demographic and clinical data of the study population (*n* = 178)

	Total, *N* = 178	GSW, *N* = 125	SW, *N* = 53	*P* value
Age, years	29.6 ± 11.7	28.1 ± 11.1	33.3 ± 12.3	0.006
Male	92.7% (165)	95.2% (119)	86.8% (46)	0.061
ISS mean ± SD	20.3 ± 12.7	22.5 ± 12.5	15.1 ± 11.6	<0.001
Low-grade liver injury	65.7% (117)	56.0% (70)	88.7% (47)	<0.001
Moderate liver injury	15.7% (28)	18.4% (23)	9.4% (5)	0.133
High-grade liver injury	18.5% (33)	25.6% (32)	1.9% (1)	<0.001
SBP < 90 mmHg on admission	16.3% (29)	17.9% (22)	13.5% (7)	0.472
GCS ≤ 8 on admission	11.8% (21)	12.1% (15)	11.3% (6)	0.884
Associated intra-abdominal injuries	79.8% (142)	88.0% (110)	60.4% (32)	<0.001
Diaphragm	39.3% (70)	41.6% (52)	34.0% (18)	0.340
Stomach	30.9% (55)	39.2% (49)	11.3% (6)	<0.001
Colon/Rectum	29.2% (52)	38.4% (48)	7.5% (4)	<0.001
Kidney	20.8% (37)	26.4% (33)	7.5% (4)	0.005
Small bowel	16.3% (29)	21.6% (27)	3.8% (2)	0.003
Spleen	15.2% (27)	18.4% (23)	7.5% (4)	0.065
Pancreas	12.4% (22)	15.2% (19)	5.7% (3)	0.077
Duodenum	10.7% (19)	13.6% (17)	3.8% (2)	0.052
Major vascular injury	9.0% (16)	12.0% (15)	1.9% (1)	0.040
Angioembolization	3.4% (6)	4.8% (6)	0.0% (0)	0.181
Surgical management				
Nonoperative	16.9% (30)	8.8% (11)	35.8% (19)	<0.001
Operative liver repair	16.9% (30)	21.6% (27)	5.7% (3)	0.009
Laparotomy with no liver repair	66.3% (118)	69.7% (87)	58.5% (31)	0.185

SD: STANDARD DEVIATION; GSW: GUNSHOT WOUND; SW: STAB WOUND; ISS: INJURY SEVERITY SCORE; BP: SYSTOLIC BLOOD PRESSURE; GCS: GLASGOW COMA SCALE.

Overall, 142 (79.8%) patients had associated intra-abdominal injuries, with the diaphragm being the most commonly injured organ (39.3%), followed by the stomach (30.9%), and the colon/rectum (29.2%) [Table 1]. Victims of a GSW had significantly more often concomitant injuries compared to patients sustaining a SW (88.0% vs. 60.4%, *P* < 0.001).

**Figure 2:**
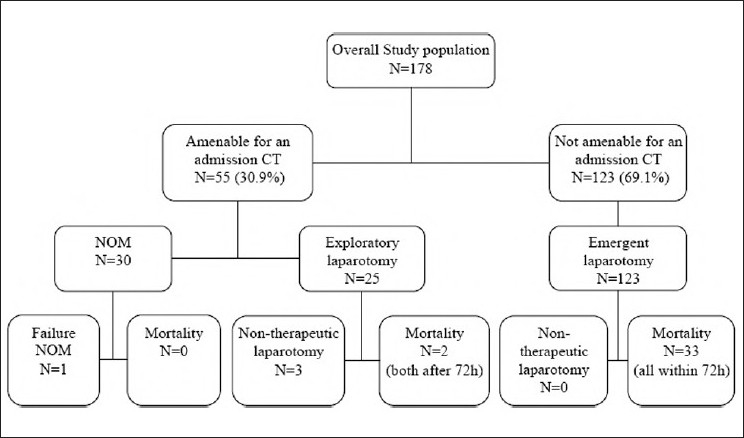
Study outline. NOM: Nonoperative management

Fifty-five of 178 (30.9%) patients were hemodynamically stable and without signs of peritonitis on admission permitting a CT scan evaluation [Figure 2]. On the basis of these CT findings, 30 (54.5%) were considered isolated liver injuries and were selected for a NOM (two patients in this group underwent angioembolisation), and 25 (45.5%) subsequently underwent laparotomy.

**Figure 3:**
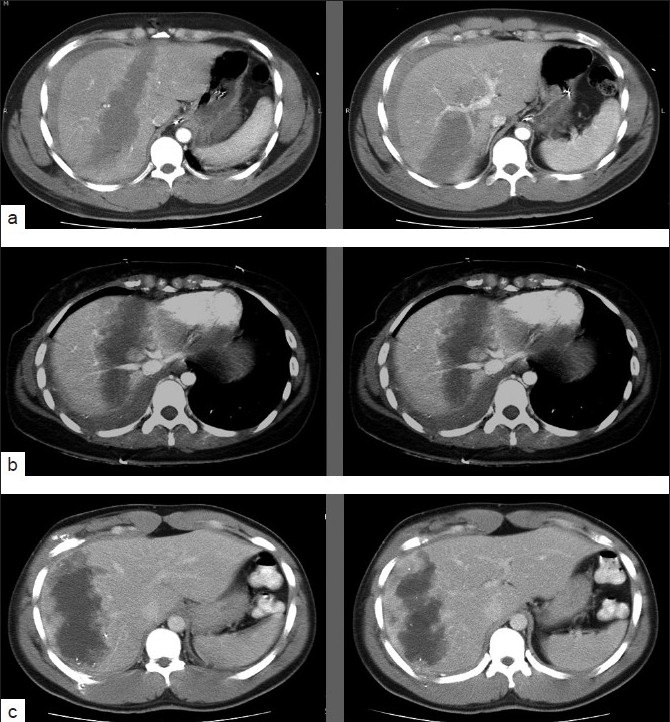
CT scans of the three patients with high-grade liver injuries treated nonoperatively (a) Patient 1, (b) Patient 2, (c) Patient 3

Of the 30 patients selected for NOM, one (3.3%) patient with a grade II liver injury failed NOM due to a missed small colonic injury after a GSW (false-negative CT-finding). The patient underwent primary repair 9 h after admission and was subsequently discharged without any postoperative complication. Overall, 16.2% (29 of 178) of all penetrating liver injuries, including six cases with grades III to V, or 80.6% (29 of 36) patients with isolated liver injuries were successfully managed nonoperatively. Three patients with high-grade injuries were treated successfully nonoperatively [Figure 3].

Twenty-five of the 55 patients (45.5%) amenable for an initial CT underwent a subsequent laparotomy due to significant CT-findings (free intraperitoneal gas, bowel wall thickening, and active contrast extravasation) [Figure 2]. Three of these laparotomies were considered nontherapeutic (false-positive CT-findings). Sensitivity and specificity of the admission CT to predict a positive laparotomy (hollow viscus injury, hemorrhage requiring surgical control) was 95.7% and 90.6%, respectively. Sensitivity and specificity of free intraperitoneal air predicting a hollow viscus injury in this group of patients was 88.2% and 50.0%, respectively.

Overall, 123 of 178 patients (69.1%) were taken emergently to the operating room without preoperative CT due to signs of peritonitis (*n* = 85, 47.8%), hypotension (*n* = 29, 16.3%), or unevaluable abdominal exam because of a GCS ≤ 8 (*n* = 9, 5.1%). Of these patients, 31 (25.2%) required surgical control for liver-related hemorrhage, and 92 patients (74.8%) underwent surgical repair of associated injuries. No nontherapeutic laparotomy was performed in this group. The overall incidence of nontherapeutic laparotomies in the group of 148 patients who underwent laparotomy was 2.0% (three cases).

### Liver-related complications and the role of routine follow-up CT

Thirty-three of 178 patients (18.5%) died within the first 72 h and were excluded from this analysis. Twelve patients died because of massive bleeding from the liver and the remaining 21 due to other major associated injuries. Of the remaining 145 patients, 88 (60.7%) patients did not have a follow-up CT because of low-grade injuries (78 patients), violation of the protocol (eight patients with grade III injuries, one patient with a grade IV injury), or transfer to another facility (one patient after angioembolisation of a grade IV injury). A total of 23.5% (24 of 102) of patients with a low-grade injury, 70.0% (19 of 27) of patients with a moderate injury, and 87.5% (14 of 16) of victims with severe LI had a CT follow-up.

Follow-up CT diagnosed 33 liver-related complications in 25 patients [Table 2]. Of those, 12 patients required intervention by means of percutaneous drainage. The overall complication rate among low-grade, moderate, and high-grade liver injuries was 7.8%, 37.0%, and 46.7%, respectively [Table 3]. Liver-related complications occurred more often after GSW compared to SW (24.0% vs. 4.1%, *P* < 0.001) [Table 2].

**Table 2:** List of liver-related complications

Complication	Total, *N* = 145	GSW, *N* = 96	SW, *N* = 49	Required intervention, % (*n*)
Fluid collection	11 (7.6)	11 (11.5)	0	27.3 (3)
Hematoma	8 (5.5)	6 (6.3)	2 (4.1)	12.5 (1)
Liver abscess	5 (3.4)	5 (5.2)	0	100.0 (5)
Bilioma	5 (3.4)	5 (5.2)	0	60.0 (3)
Liver necrosis	4 (2.8)	4 (4.2)	0	0.0 (0)
Total number of patients with liver-related complicationsa	25 (17.2)	23 (24.0)	2 (4.1)	48.0 (12)

GSW: GUNSHOT WOUND; SW: STAB WOUND; ^A^EIGHTEEN PATIENTS HAD ONE COMPLICATION, SIX PATIENTS HAD TWO COMPLICATIONS, AND ONE PATIENT HAD THREE LIVER-RELATED COMPLICATIONS (33 COMPLICATIONS IN 25 PATIENTS); FIGURES IN PARENTHESIS ARE IN PERCENTAGE

**Table 3:** Liver-related complications in relation to the grade of liver injury and the treatment modality

Grade of liver injury	Overall, % (*n* = 145)	NOM, % (*n* = 30)	Laparotomy with liver repair % (*n* = 24)	Laparotomy without liver repair % (*n* = 91)
Low (*n* = 103)	7.8 (8/103)	4.2 (1/24)	9.1 (1/11)	8.8 (6/68)
Moderate (*n* = 27)	37.0 (10/27)	0.0 (0/3)	40.0 (4/10)	43.9 (6/14)
High (*n* = 15)	46.7 (7/15)	0.0 (0/3)	66.7 (2/3)	55.6 (5/9)
Total (*n* = 145)	17.2 (25/145)	3.3 (1/30)	29.2 (7/24)	18.7 (17/91)

NOM: NONOPERATIVE MANAGEMENT

The mean time to diagnosis of liver-related complications was 11.6 ± 8.5 days postadmission. In 18 of the 25 (72.0%) patients, the complications were diagnosed within the first 14 days. The latest complication that occurred was liver necrosis 35 days after laparotomy, liver suturing and angioembolisation of a grade IV injury.

The highest rate of liver-related complications was observed in patients with moderate-to-high-grade injuries requiring laparotomy (17 of 36, 47.2%), of which 9 (52.9%) required an intervention. The complications diagnosed in this subgroup of patients consisted of nine fluid collections, six hematomas, five bilomas, three liver necrosis, and two liver abscesses. Six patients with liver-related complications requiring intervention were symptomatic (fever, abdominal pain, and elevation of white blood cell counts). In 3 of these 36 patients (8.3%), asymptomatic bilomas were noted. No liver-related complications occurred in the six patients with moderate and high-grade injuries treated nonoperatively [Table 3].

## DISCUSSION

Selective NOM of *blunt* liver injuries has become the only acceptable standard of care. However, following penetrating trauma, particularly in victims of GSW, exploratory laparotomy has remained the standard of care for many decades. This policy was challenged in recent studies that supported selective NOM in clinically evaluable and hemodynamically stable patients without peritonitis.[1–4,6–8] Thus, at LAC + USC Medical Center, all evaluable patients following penetrating injury to the abdomen, who are hemodynamically stable and have no signs of peritonitis, undergo a contrast-enhanced CT scan evaluation of the abdomen. In the presence of radiological signs of hollow viscus perforation or evolving hemodynamic instability or peritonitis undergo exploratory laparotomy. The severity of the LI *per se* is not considered as a contraindication for NOM.

Contrast-enhanced CT scan can be of value in selecting the optimal treatment for the patient with suspected LI by providing information about the severity of the liver lesion, the presence of active bleeding or false aneurysms and associated thoracoabdominal injuries. The experiences with the admission CT in patients sustaining a penetrating LI, however, are limited, because the majority of these patients are not amenable for a preoperative advanced imaging. Likewise, according to the data presented in the current series, only a third of patients underwent a CT on admission. For these patients, the accuracy of CT scan in identifying patients requiring surgical intervention has not been evaluated in the literature. This study has shown that the CT scan has a sensitivity of almost 96% and a specificity of 91% in predicting the need for operative intervention. Combined with clinical examination, the sensitivity reaches 99% but specificity remains at 91%. Of the 30 patients who were selected for NOM, only one (3.3%) significant injury was missed by the initial physical examination and the contrast CT scan. However, in this series, the value of the initial CT evaluation to rule out a hollow viscus injury was poor (specificity 50%). Therefore, serial clinical examinations of the abdomen and white blood cell count monitoring are critical in all patients with penetrating abdominal trauma selected for NOM irrespective of the initial clinical examination or CT scan findings.

The severity of LI on the CT scan does not reliably predict the need for operative management of the liver. Of the 61 patients with grades III and IV liver injuries, 6 (9.8%) were successfully managed nonoperatively without complications. On the other hand, only three patients with isolated grades I and II liver injuries on CT scan underwent laparotomy.

The role of follow-up CT scan evaluation in blunt liver trauma is controversial. Some studies recommend routine CT scanning while others support CT evaluation only in symptomatic patients.[11,13–15] In this study the number of patients who underwent a follow-up CT increased with increasing severity of LI. Only about a quarter of low-grade injuries, but almost 90% of patients with high-grade injuries had a follow-up CT. This practice was based on the individual trauma surgeon’s judgment. In general, recommendations on the need for a follow-up CT after blunt, and in particular after penetrating LI, are scarce. In a previous study from our center, it was reported that nearly 50% of patients with moderate or severe liver injuries managed operatively, developed a postoperative liver-related complication. About 12% (2 of 17) of asymptomatic patients had a significant complication requiring intervention.[10] This study confirms this finding for the operatively managed patients. Overall, 17 of 42 (40.5%) patients with grades III and V injuries developed liver-related complications.

The role of follow-up CT scan in minor liver injuries remains uncertain. Although this study showed only one clinically nonsignificant complication in an asymptomatic patient, no solid conclusion can be made because only 24 of 102 (23.5%) patients in this group underwent follow-up CT evaluation. Further prospective investigation is warranted to investigate the role of follow-up imaging in patients with low-grade penetrating liver injuries.

## CONCLUSION

Two-thirds of patients with penetrating liver injuries require emergent laparotomy due to hemodynamic instability, peritonitis, or unevaluable abdomen and therefore are not amenable for preoperative CT evaluation. The majority of these patients require surgical intervention for concomitant injuries. One-third of patients with penetrating liver injuries are amenable for an admission CT, which reliably predicts successful NOM. However, hollow viscus injuries may remain undetected, and therefore, serial examinations of the abdomen and monitoring of white cell count are critical in all patients selected for NOM, irrespective of the initial clinical examination and CT scan findings. Nevertheless, more than 80% of isolated penetrating liver injuries can safely be managed nonoperatively.

In moderate-to-high-grade penetrating liver injuries, a mandatory in-hospital follow-up CT is advocated due to the high incidence of liver-related complications, which may be asymptomatic.

## References

[1] Demetriades D, Rabinowitz B, Sofianos C (1986). Non-operative management of penetrating liver injuries: A prospective study. Br J Surg.

[2] Demetriades D, Hadjizacharia P, Constantinou C, Brown C, Inaba K, Rhee P (2006). Selective nonoperative management of penetrating abdominal solid organ injuries. Ann Surg.

[3] Demetriades D, Gomez H, Chahwan S, Charalambides K, Velmahos G, Murray J (1999). Gunshot injuries to the liver: The role of selective nonoperative management. J Am Coll Surg.

[4] Omoshoro-Jones JA, Nicol AJ, Navsaria PH, Zellweger R, Krige JE, Kahn DH (2005). Selective non-operative management of liver gunshot injuries. Br J Surg.

[5] Velmahos GC, Demetriades D, Toutouzas KG, Sarkisyan G, Chan LS, Ishak R (2001). Selective nonoperative management in 1,856 patients with abdominal gunshot wounds: Should routine laparotomy still be the standard of care?. Ann Surg.

[6] DuBose J, Inaba K, Teixeira PG, Pepe A, Dunham MB, McKenney M (2007). Selective non-operative management of solid organ injury following abdominal gunshot wounds. Injury.

[7] Renz BM, Feliciano DV (1995). Gunshot wounds to the liver: A prospective study of selective nonoperative management. J Med Assoc Ga.

[8] Navsaria PH, Nicol AJ, Krige JE, Edu S (2009). Selective nonoperative management of liver gunshot injuries. Ann Surg.

[9] Velmahos GC, Constantinou C, Tillou A, Brown CV, Salim A, Demetriades D (2005). Abdominal computed tomographic scan for patients with gunshot wounds to the abdomen selected for nonoperative management. J Trauma.

[10] Demetriades D, Karaiskakis M, Alo K, Velmahos G, Murray J, Asensio J (2003). Role of postoperative computed tomography in patients with severe liver injury. Br J Surg.

[11] Cuff RF, Cogbill TH, Lambert PJ (2000). Nonoperative management of blunt liver trauma: The value of follow-up abdominal computed tomography scans. Am Surg.

[12] Tinkoff G, Esposito TJ, Reed J, Kilgo P, Fildes J, Pasquale M (2008). American Association for the Surgery of Trauma Organ Injury Scale I: Spleen, liver, and kidney, validation based on the National Trauma Data Bank. J Am Coll Surg.

[13] Pachter HL, Knudson MM, Esrig B, Ross S, Hoyt D, Cogbill T (1996). Status of nonoperative management of blunt hepatic injuries in 1995: A multicenter experience with 404 patients. J Trauma.

[14] Croce MA, Fabian TC, Menke PG, Waddle-Smith L, Minard G, Kudsk KA (1995). Nonoperative management of blunt hepatic trauma is the treatment of choice for hemodynamically stable patients. Results of a prospective trial. Ann Surg.

[15] Kozar RA, Moore FA, Cothren CC, Moore EE, Sena M, Bulger EM (2006). Risk factors for hepatic morbidity following nonoperative management: Multicenter study. Arch Surg.

